# Achieving Narrowband and Stable Pure Blue Organic Light‐Emitting Diodes by Employing Molecular Vibration Limited Strategies in the Extended π‐Conjugated Indolocarbazole Skeleton

**DOI:** 10.1002/advs.202410479

**Published:** 2025-01-13

**Authors:** Zhiheng Wang, Xuming Hu, Zhiping Yan, Jie Liang, Xiaoxian Song, Qishen Chen, Hai Bi, Yue Wang

**Affiliations:** ^1^ Department of Materials Science and Technology Ji Hua Laboratory Foshan Guangdong 528200 P. R. China; ^2^ Jihua Hengye Electronic Materials Co. Ltd. Foshan Guangdong 528200 P. R. China; ^3^ State Key Laboratory of Supramolecular Structure and Materials College of Chemistry Jilin University Changchun 130012 P. R. China

**Keywords:** π‐conjugated indolocarbazole, molecular vibration limited strategy, narrowband emission, organic light‐emitting diode

## Abstract

B‐ and N‐heterocyclic fluorophores have reveal promising efficiency in blue organic light‐emitting diodes (OLEDs) with small full‐width‐at‐half‐maximum (FWHM). However, their structural determinants for spectral broadening and operating stability are still needed to be investigated in further. Herein, a novel multi‐N‐heterocycles Diindolo[3,2,1jk:3′,2′,1′jk]dicarbazole[1,2‐b:4,5‐b] (DIDCz) is proposed to manipulate the emission color toward pure blue region by extending π‐conjugation of the N‐π‐N bridge. By utilizing computed spectral technique, interrelationships between indolocarbazole (ICz)‐cyclization sites and spectral broadening are defined. Molecular backbone modifications involving optimized 2,3,6,7‐ICz cyclization and steric hindrance substituents are conducive to restricting swing of peripheral bonds and stretching resonances of the polycyclic aromatic hydrocarbon (PAH) frameworks, thereby contributing to the reduction of shoulder emission peaks. Consequently, the DIDCz‐based chromophore exhibited narrowband blue emission with a FWHM of only 15 nm, achieving highly efficient (external quantum efficiencies of 8.87% in triplet–triplet fusion fluorescence and 22.3% in sensitized fluorescence) and long‐term (95% of the initial luminance of 1000 cd m^−2^, T95 = 1545 h) electroluminescence performances, showing one of the most narrowband and stable blue OLEDs among the reported PAH chromophores. The current achievements offer a new perspective to manage spectral broadening precisely based on the molecular vibration limiting technique.

## Graphical abstract

By employing the molecular vibration limited strategy to restrict molecular vibrations of the polycyclic aromatic hydrocarbon framework, the multi‐N‐heterocycles DIDCz‐tBu exhibited narrowband blue emission with a full‐width‐at‐half‐maximum of 15 nm, achieving highly efficient (8.87% in triplet–triplet fusion fluorescence and 22.3% in sensitized fluorescence) and operational stale (T95 lifetime of 1545 h) electroluminescent performances in organic light‐emitting diodes.

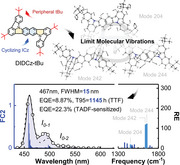

## 1 Introduction

With remarkable advancements in display technology driven by organic light‐emitting diodes (OLEDs), emitting materials with narrowband spectra have become indispensable to meet the escalating demands for color purity and luminous efficiency. Traditional methods to achieve narrow emission involve utilizing optical microcavity of light at a specific wavelength and coating band‐pass color filters.^[^ ^1^ ^]^ The latter approach filters out unnecessary light energies, leading to a significant out‐coupling efficiency loss in the electroluminescent (EL) cell. To address this issue, it is crucial to designing small full‐width‐at‐half‐maximum (FWHM) chromophores with negligible should peaks. At the same emission peak, narrower FWHMs manifests smaller CIEy (and higher blue index, current efficiency/CIEy) and relatively lower 0‐0 transition energies due to the red‐shift for spectral onset.^[^ ^2^ ^]^ In other words, both the driving voltage and the operational stability of blue OLEDs should be improved, as degradation rates are highly dependent on the excited state energy. Due to the difficulty in managing large bandgap fluorophores with efficient transition dipoles, molecular design for color‐saturated, high photoluminescence quantum yield (PLQY) and operational stable blue emitters are still scarce and challenging.^[^ ^3^, ^4^, ^5^, ^6^ ^]^

The spectral broadening of molecular skeletons is determined by structural relaxations and vibration couplings of excited states. When the molecular conformation in excited states is closely mirrors that of the ground state, energies of vibronic couplings are almost equal with negligible reorganization energies (*∆E*s), resulting in a narrow 0‐0 vibration peak without spectral tails. An increase of structural changes between ground and excited states leads to broader FWHMs, owing to the growth of ∆*E*s and structural relaxations.^[^ ^7^ ^]^ With advancements in chemical computation, a certain vibronic coupling corresponds to specific molecular vibration features. Typically, molecular vibrations at low vibrational frequencies (0‐1300 cm^−1^) and high vibrational frequencies (1300‐1800 cm^−1^) govern the 0‐0 vibration peak and multitudinous shoulder peaks respectively.^[^ ^8^ ^]^ To reduce unnecessary shoulder peaks, molecular vibrations at high vibrational frequencies should be confined carefully, while an ideal spectral shape should also consist of a narrow FWHM 0‐0 peak. To date, only few research studies of polycyclic aromatic hydrocarbon (PAH) emitters have systematically concluded the ascriptions between molecular vibration characteristics and multitudinous vibronic couplings.^[^ ^9^, ^10^, ^11^, ^12^, ^13^ ^]^ Those molecular vibration modes with large *∆E*s in the reorganization energy distribution are uncertainly contributed to vibrational emission.

Conventional blue PAH derivatives, including anthracene, pyrene, naphthalene, have achieved high efficiency and long‐term operating lifetimes owing to the large transition dipole strength and stable bond dissociation energies.^[^ ^14^, ^15^, ^16^, ^17^, ^18^ ^]^ These emissive skeletons typically exhibit a narrow 0‐0 emission peak, accompanied by multiple shoulder peaks. In 2018, Hatakeyama et al. reports a multiple‐resonance (MR) type boron/nitrogen (B/N) emitter, giving suppression of major stretching vibrations to reduce shoulder peaks and leading to a FWHM of 18 nm in the ν‐DABNA‐based devices. Based on this concept, a series of narrowband blue PAH emitters have been developed by embedding B/N,^[^ ^19^, ^20^, ^21^, ^22^, ^23^, ^24^, ^25^, ^26^ ^]^ boron/oxygen,^[^ ^27^, ^28^, ^29^, ^30^ ^]^ nitrogen/carbonyl (N/C═O),^[^ ^31^, ^32^, ^33^ ^]^ indolocarbazole (ICz)^[^ ^2^, ^34^, ^35^, ^36^ ^]^ emitting frameworks. Some molecular backbone modifications, including steric hindrance chains (eg. tertiary butyl, trimethylbenzene),^[^ ^20^, ^37^, ^38^ ^]^ five‐ or six‐membered heterocycles,^[^ ^39^, ^40^ ^]^ intramolecular spatial interlock^[^ ^41^ ^]^ etc., have been proposed in previous reports, showing small FWHMs of 30 nm in general. However, only few studies have identified molecular vibration characteristics that primarily contribute to spectral broadening. The progression of molecular vibration changes in relation to molecular backbone modifications remains unclear yet. Therefore, it is challenging to determine the exact modification sites to restrain vibrational emission effectively. Eventually, molecular design strategies for constructing narrowband chromophores still rely on empirical evidence.

More recently, Lee et al. reports a new N‐atom embedded PAH skeleton indolo[3,2,1‐jk]carbazole that exhibited near violet emission with a FWHM of only 14 nm.^[^ ^42^ ^]^ Duan's group assembles an blue‐violet fluorophore (453 nm) of 8′,18′‐ditert‐butyl‐2′,12′‐dimethyldispiro[fluorene‐9,10′‐dibenzo[2,3:5,6]indolizino[1,8‐ab]indolo[3,2,1‐de]acridine‐20′, 9″‐fluorene] (pSFIAc2),^[^ ^2^ ^]^ achieving a small FWHM of 19 nm and T80 (lifetime to 80% of the initial luminance) of ≈230 h at 2000 cd m^−2^. Other derivatives with ICz‐based cyclization exhibit narrow EL spectra but still emitted too violet coordinates. To regulate the emission peak to pure blue region, a common strategy is introducing electronic push/pull substituents into the PAH cores. These substituents not only control the ICT interaction of frontier molecular orbitals, but also bring new structural relaxations, inducing new vibrational peaks into emission spectra. For example, Wei et al. successfully red‐shift the CIEy value to 0.10 by para‐substituting diphenylamines into the ICz‐PAH skeleton, but the emission shape becomes relatively broad with a FWHM of 41 nm.^[^ ^43^ ^]^ Therefore, it is urgently needed to design an intrinsic narrowband and efficient blue‐emitting skeleton without further electronic push/pull modifications.

In this work, we developed a narrowband and efficient blue emitting framework by integrating ICz‐based groups into the naphthyl PAH unit, resulting in a novel narrowband chromophore of Diindolo[3,2,1jk:3′,2′,1′jk]dicarbazole[1,2‐b:4,5‐b] (DIDCz). By utilizing advances of quantum chemical calculations, the relationships between ICz‐cyclization sites and the spectral profiles were elucidated, choosing the optimal 2,3,6,7‐ICz cyclization pattern for constructing a narrowband blue chromophore. The evolution of molecular vibration changes in response to structural modifications can be concluded among DIDCz‐based emitters. Consequently, strategies involving five‐membered heterocycle cyclization and steric hindrance peripheral groups effectively constrain inherent molecular resonances (in‐plane stretching and swing behaviors) of the PAH skeleton and peripheral bonds. Attributed to the π‐conjugated N‐naphthyl‐N (N‐*Na*‐N) skeleton, a red‐shifted ultrapure blue emission with bonding/antibonding characters was achieved. the blue emitter DIDCz‐tBu afforded 96.5% in PLQYs and small FWHM of 15 nm, accompanied by reduced spectral trails. The pure blue OLEDs demonstrated remarkable external quantum efficiencies (EQEs) of 8.87% in the triplet–triplet fusion (TTF) fluorescence and 22.3% in the thermally activated delayed fluorescent‐sensitized fluorescence (TSF), giving long‐lived operating T95 lifetime (95% of initial luminance of 1000 cd m^−2^) in TTF‐based devices.

## 2 Results and Discussion

### 2.1 Controlling Spectral Broadening of Blue Fluorophores

**Figure 1:**
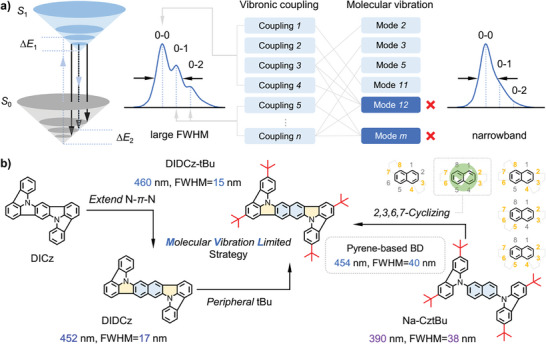
a) The introduction of controlling vibrationally resolved spectrum profiles for the emitting fluophosphors. b) Molecular vibration limited strategies and ICz‐cyclization optimizations for synthesizing narrowband blue chromophores with a para‐positioned N‐*Na*‐N central bridge.

ICz‐based fluorophores have demonstrated promising quantum yields and narrow FWHMs due to their increased rigidity frameworks and short‐range MR frontier orbitals. Among them, Indolo[3,2,1‐jk]indolo[1′,2′,3′:1,7]indolo[3,2‐b]carbazole (DICz) with peripheral tert‐butyl substituents, named pICz, exhibits a bluish violet emission at 441 nm and a FWHM of 18 nm.^[^ ^43^ ^]^ The DICz skeleton can be regarded as a para‐positioned N‐phenyl‐N (N‐*Ph*‐N) bridge in the central core. These N‐heterocyclic chromophores are limited by the ultraviolet emission of the short‐conjugated ICz unit, and it is challenging to tune the emission color beyond deep blue. An optional strategy to red‐shift emission peak is introducing electron‐donating groups on the ICz skeleton. However, this may further induce unnecessary vibrational peaks into emission spectra, resulting in a color purity reduction. To address this issue, we extend the π‐conjugated phenyl group to a naphthyl PAH group, synthesizing a novel chromophore of DIDCz, with a para‐positioned N‐*Na*‐N in the central bridge. Hence, the pristine DIDCz skeleton is expected to provide pure blue emission color without further electron‐donating/withdrawing modifications. The spectral shape control of DIDCz‐based fluorophores was investigated by combining quantum chemical calculations. Generally, the spectral shape of molecular skeleton is determined by multitudinous vibronic couplings that associated with specific molecular vibration modes. Based on this relation, narrowband spectra with confined vibrational shoulder peaks (0–1, 0–2, etc. peaks) could be achieved when molecular vibrations for spectral broadening are spatially constrained (**Figure** **1**a). To validate this concept, we introduce molecular vibration limited strategies to acquire narrowband blue emitters through ICz‐cyclization and attaching peripheral steric hindrance substituents. The interrelationships between ICz‐cyclization sites and spectral shape manipulations were discussed to distinguish the optimal cyclization pattern among the N‐*Na*‐N‐based frameworks (Figure 1b).

According to the molecular design for narrowband blue chromophores, vibrational spectra, and reorganization energies were first computed using sum‐over‐states (SOS) and evc methods in the Molecular Materials Property Prediction Package (MOMAP) program.^[^ ^44^, ^45^, ^46^ ^]^ The conformation of ground (*S*_0_) and singlet (*S*_1_) states has been optimized by the B3LYP/svp level in advance. First, the emission spectral profiles of different ICz‐cyclization sites in the construction of N‐*Na*‐N‐based skeleton were computed using the SOS method. As shown in Figure S5a (Supporting Information), the 2,3,6,7‐ICz cyclization (DIDCz) candidate displayed an inherently ultrapure blue emission with reduced 0–1 and 0–2 vibrational peaks, in contrast to 3,4,7,8‐ICz cyclization and 2,3,4,5,6,7‐ICz cyclization candidates. The 2,3,4,6,7,8‐ICz cyclization and 2,3,5,6,7‐ICz cyclization skeletons also revealed less pronounced spectral trails but their emission color has moved to sky‐blue and blue‐violet region, respectively. Compared to the main emission peak (*I*_0‐0_), the emission intensities of 0–1 vibrational peak for 3,4,7,8‐ICz cyclization, 2,3,6,7‐ICz cyclization, 2,3,4,6,7,8‐ICz cyclization, 2,3,5,6,7‐ICz cyclization and 2,3,4,5,6,7‐ICz cyclization skeletons were 45.6%, 23.0%, 24.2%, 23.9% and 30.4%, respectively, demonstrating 2,3,6,7‐ICz cyclization chromophore as the optimal cyclization pattern. Additionally, introduction of a N‐*Na*‐N central bridge instead of N‐*Ph*‐N achieved a more rigid framework with reduced vibrational emission (Figure S5c, Supporting Information). As a result, the DIDCz skeleton (17 nm) exhibited a smaller FWHM than the pristine DICz (20 nm) in calculated spectral profiles. The emission peak of DIDCz red‐shifted to 458 nm, as compared to 439 nm in DICz. Therefore, our proposed DIDCz‐based emitters are capable of producing ultrapure blue emission.

After determiningthe optimal ICz‐cyclization pattern, spectral profile manipulations of the 2,3,6,7‐ICz cyclization and peripheral tertiary butyl substituents were investigated. As illustrated in Figure S1a–c (Supporting Information), the pristine DIDCz core showed narrowband calculated absorption and emission spectra (corresponding to *S*_0_‐*S*_1_ and *S*_1_‐*S*_0_ transitions, respectively). The emission intensities of 0–1 and 0–2 vibrational peaks (denoted as *I*_0‐1_ and *I*_0‐2_) were 23.0% and 2.6%, respectively, as compared to the main emission peak (*I*_0‐0_). Upon the introduction of steric hindrance substituents, emission shoulder peaks of DIDCz‐tBu further reduced to 19.3% and 1.7%, suggesting that unnecessary spectral trails can also be controlled by peripheral groups. Conversely, non‐polycyclic Na‐tBuCz emitters exhibited significant spectral broadening with non‐vibrational features. The five‐membered ICz‐cyclization of polycyclic‐ICz offers significant contributions to constrain structural relaxations, thereby enhancing rigidity of the molecular framework. For further comparisons, the calculated spectral profile of a commercial pyrene‐based blue emitter, N^1^,N^6^‐bis(dibenzo[*b*,*d*]furan‐4‐yl)‐3,8‐diisopropyl‐N^1^,N^6^‐bis(4‐isopropylphenyl)pyrene‐1,6‐diamine (BD) was also investigated, showing a broader emission shape with more pronounced shoulder peaks (*I*_0‐1_ = 33.2% and *I*_0‐2_ = 5.0%). To validate simulated outcomes, UV‐vis absorption and photoluminescence (PL) spectra of DIDCz‐based emitters were measured in toluene, showing high agreement with the theoretical results. Among these emitters, the DIDCz‐tBu fluorophore achieved pure blue emission at 463 nm with a narrow FWHM of only 15 nm, in contrast to 40 nm for the pyrene‐based BD dye. This represents one of the most color‐saturated achievement among the reported ICz‐based emitters (Table S6, Supporting Information).

Structural relaxations between *S*_0_ and *S*_1_ states can be quantized by the distribution of reorganization energies in further. Generally, low vibrational frequency (0–1300 cm^−1^) and high vibrational frequency (*ω =* 1300‐1800 cm^−1^) molecular vibrations dominate the 0‐0 spectral peak and shoulder peaks, respectively.^[^ ^7^ ^]^ Total *∆E*s for 3,4,7,8‐ICz cyclization, 2,3,6,7‐ICz cyclization, 2,3,4,6,7,8‐ICz cyclization, 2,3,5,6,7‐ICz cyclization and 2,3,4,5,6,7‐ICz cyclization frameworks were 265 meV, 162 meV, 161 meV, 201 meV and 227 meV, respectively. These values correlated with the calculated spectral profiles accurately. For the DIDCz‐based emitters, total *∆E*s of DIDCz‐tBu, DIDCz and Na‐tBuCz candidates were 154 meV, 162 meV and 455 meV, which was in agreement with the simulated results. The total *∆E*s of DIDCz‐tBu was also smaller than the pristine DICz skeleton (258 meV) and the pyrene‐based BD emitter (235 meV). Hence, a broader emission shape with distinct vibrational peaks was expected (Figures S5c and S10c, Supporting Information). The *∆E* distribution characteristics were strongly influenced by cyclization patterns and molecular backbones, making challenges to distinguish the evolution of molecular vibration changes. With respect to *∆E* distributions within the vibrational frequency range of 1300–1800 cm^−1^, the primary structural relaxations of three N‐*Na*‐N‐based molecules were located at 1350–1540 cm^−1^ and 1620–1730 cm^−1^ regions. The employment of ICz‐based cyclization at N‐*Na*‐N bridge significantly reduced *∆E*s in the 1350–1540 cm^−1^ region, while attaching additional peripheral substituents to the polycyclic PAH framework further diminished resonances of multi‐N‐heterocycles to a certain extent. It is worth noting that, for the narrowband emitter DIDCz‐tBu, *∆E*s were highly concentrated at the vibrational frequency of 1300–1800 cm^−1^ (74.3%), compared to 71.6% and 48.2% for DIDCz and Na‐tBuCz emitters, respectively, suggesting that unnecessary structural relaxations of the main emission peak have been restricted.

**Figure 2:**
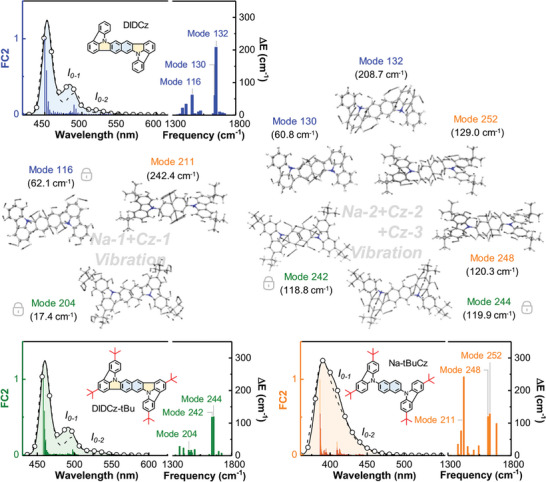
Calculated vibrationally resolved and experimental (in toluene) emission spectra of the DIDCz, DIDCz‐tBu, and Na‐tBuCz (the calculated emission peak has aligned to experimental spectra). The reorganization energy distribution of DIDCz, DIDCz‐tBu, and Na‐tBuCz emitters in the *S*_1_‐*S*_0_ transition. The evolution of molecular vibration changes that mainly contribute to the vibrational emission in the N‐*Na*‐N‐based emitters (reorganization energies of the molecular vibration mode are shown in the bracket), identifying as the *Na*‐1+*Cz*‐1 type (≈1430 cm^−1^) and the *Na*‐2+*Cz*‐2 or *Na*‐2+*Cz*‐3 type (≈1630 cm^−1^) vibrations.

With similar *∆E* distribution characteristics in DIDCz‐based emitters, relationships between vibronic couplings and molecular vibrations characteristics can be further delineated to comprehend the evolution of molecular vibration behaviors in response to structural modifications (Figures S3 and S4, Supporting Information). The molecular vibration mode of 211 (*ω* = 1425.46 cm^−1^, 39.6%), 248 (*ω* = 1617.45 cm^−1^, 16.5%) and 252 (*ω* = 1631.59 cm^−1^, 13.1%) were mainly donated to 0–1 and 0–2 emission peaks of the non‐polycyclic Na‐tBuCz. The vibration mode 211 exhibited an in‐plane stretching of the naphthyl PAH unit (named as the *Na*‐1 vibration) and an in‐plane swing of peripheral C‐H bonds at carbazole groups (named as the *Cz*‐1 vibration) (**Figure** **2**). Molecular vibrations at ≈1630 cm^−1^ (mode 248 and mode 252) showed another in‐plane C─C and C─H bond stretching feature of the naphthyl unit (*Na*‐2 vibration) and structural relaxations of carbazole groups (*Cz*‐2 vibration). Upon the introduction of ICz‐cyclization, *Na*‐1 type vibrations at ≈1430 cm^−1^ were reduced to 15.4% for DIDCz (mode 116) and 4.5% for DIDCz‐tBu (mode 204) by the rigid polycyclic DIDCz. The reason for molecular vibration controls is that in‐plane five‐ring cyclization spatially restrains inherent stretching of the naphthyl component, as compared to the torsional conformation of Na‐tBuCz molecules. Moreover, the *Na*‐2 type vibrations emerged as major contributors for naphthyl relaxations (mode 130 and 132 for DIDCz, mode 242 for DIDCz‐tBu), while the in‐plane oscillation of ICz changed to another molecular resonance behavior (*Cz*‐3 vibration) due to the ICz‐PAH cyclization. The attachment of tertiary butyl groups to the DIDCz framework moderates the *Na*‐2 type and *Cz*‐3 type vibrations to a certain extent without introducing new molecular vibrations.

### 2.2 Photophysical Properties of the DIDCz‐Based Emitters

**Figure 3:**
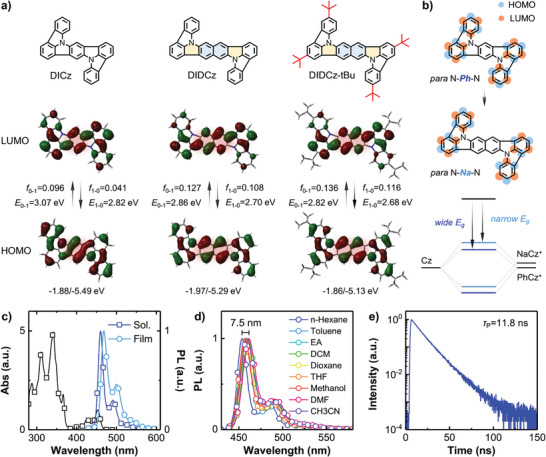
a) HOMO and LUMO distributions, *f*, and energy gaps (*E*_g_s) of the DICz, DIDCz, and DIDCz‐tBu emitters in the *S*_0_‐*S*_1_ and *S*_1_‐*S*_0_ transitions. b) The MR frontier orbitals of the DIDCz‐based skeletons with a para‐positioned N‐*Ph*‐N bridge for DICz and N‐*Na*‐N bridge for DIDCz. The formation of delocalized excited states is shown below. c) UV−vis absorption (in toluene) and PL spectra (in toluene and 1% DIDCz‐tBu:BH films) of the DIDCz‐tBu. d) PL spectra of the DIDCz‐tBu in different solvents with various polarities. e) Transient PL in the 1% DIDCz‐tBu:BH blended film.

To understand photophysical properties of the designed emitters, density functional theory (DFT) and time‐dependent‐DFT (TD‐DFT) calculations using B3LYP/svp level were performed. Frontier molecular orbital (FMO) of the lowest unoccupied molecular orbital (LUMO) and the highest occupied molecular orbital (HOMO) exhibited short‐range MR distributions on peripheral phenyls of the DIDCz framework (**Figure** **3**a). It is worth noting that π‐conjugated extension for the para‐positioned central bridge has minimal effects on MR distributions. On the central PAH bridge, π‐orbitals with bonding/antibonding characters were observed in both DICz and DIDCz skeletons. Compared to DICz, more alternate bonding/antibonding distributions were performed across the para‐positioned N‐*Na*‐N bridge, generating a stronger electronic coupling of the central PAH bridge to narrow delocalized excited states (Figure 3b). Molecular orbital distribution characters were also confirmed by the natural transition orbital (NTO) analysis in Figure S7 (Supporting Information). Therefore, the calculated HOMO of DIDCz was shallower than that of DICz, and the bandgap (*E*_g_) of *S*_1_‐*S*_0_ transitions red‐shifted to pure blue region of 2.70 eV (459 nm). After the introduction of tertiary butyl substituents, *E*_g_ of DIDCz‐tBu maintained at 2.68 eV (462 nm) due to the negligible distribution of FMO s on steric hindrance groups. To further evaluate electronic transition properties between *S*_0_ and *S*_1_ states, the oscillator strength (*f*) of the *S*_1_‐*S*_0_ transition increased to 0.108 and 0.116 in DIDCz and DIDCz‐tBu molecules, compared to only 0.041 for the DICz. The development of transition dipole strengths is attributed to π‐orbitals across the extended N‐*Na*‐N bridge, leading to a larger FMO overlap.

The optimized structure of the DIDCz backbone in both *S*_0_ and *S*_1_ states showed planar conformations with five‐membered interlocked structures (Figure S8, Supporting Information), which avoided possible in‐plane stretching vibrations. Attaching additional tertiary butyl substituents offers sterically hindered to disrupt intermolecular π‐π interactions and aggregation quenching. Generally, bond length changes between optimized structures of *S*_0_ and *S*_1_ states can in somehow illustrate structural relaxations of the vibrational spectrum. The bond length changes were in accord with those reorganization energies. As results in Figure S9 (Supporting Information), bond length changes of both DIDCz (0.0046 Å) and DIDCz‐tBu (0.0024 Å) were smaller than non‐cyclized Na‐tBuCz (0.0055 Å), confirming that cyclized central PAH bridge with five‐membered ICz heterocycles increases rigidity of the DIDCz skeleton and confines possible structural relaxations.

As narrowband blue fluorophores have been achieved by employing molecular vibration limited strategy, the involved materials were synthesized as depicted in Scheme S1 (Supporting Information). Following several conventional reaction steps and sublimation purifications, DIDCz‐tBu could be obtained in high purity of 99.86% (Figure S19, Supporting Information). The precise chemical structure of the involved molecules was fully characterized using the NMR spectroscopy and the mass spectrometry (Figures S20‐S26, Supporting Information). DIDCz‐tBu displayed excellent thermal property with 5% weight‐loss decomposition temperature (*T*_d_) of 508 °C. The glass transition and melting point were not observed during differential scanning calorimetry analysis (Figure S5, Supporting Information). As revealed by cyclic voltammetry (CV) measurements, the oxidation potential peaks at 0.44 V were observed in DIDCz‐tBu (Figure S6, Supporting Information), of which the HOMO level was estimated to be ‐5.36 eV (**Table** **1**).

**Table 1:** Photophysical properties of the DIDCz‐tBu and BD emitters.

Compound	*λ*_Abs_ ^a)^	*λ*_PL_	FWHM	*S*_1/_ *T*_1_ ^c)^	*Φ*_PL_ ^b)^	*τ*_p_ ^b)^	*k*_r_ ^d)^	HOMO^e)^	LUMO^e)^
	(nm)	(nm)	(nm)	(eV)	(%)	(ns)	(10^7^ s^−1^)	(eV)	(eV)
DIDCz‐tBu	402, 426, 453	460^a)^/468^b)^	15^a)^/20^b)^	2.73/2.39	96.5	11.8	8.2	−5.36	−2.70
BD	382, 408, 431	454/462	40/48	–	94.4	3.4	27.7	−5.11	−2.37

^a)^ Measured in toluene at room temperature;

^b)^ Measured in 1% DIDCz‐tBu:BH blended films;

^c)^ The *S*_1_ and *T*_1_ state energies were determined by the onset of fluorescent and phosphorescent spectra at 77K in toluene;

^d)^ Rate constant of fluorescence radiative decay, *k*_r_ = *Ф*_PL_/*τ*_P_;

^e)^ The HOMO and LUMO energies were determined according to *E*_HOMO_ = ‐(*E*_ox_+4.8+*E*_FC+_) eV and *E*_LUMO_ = *E*_HOMO_+*E*_g_.

The computed spectra simulation and (TD)DFT calculations have expected narrowband and efficient radiations for DIDCz‐based molecules. The involved DIDCz‐tBu chromophore give promising capability to achieve high color purity and efficient blue emission. Herein, photophysical properties were investigated in order to predict the EL performance prior to device fabrications. Another high performance pyrene‐based blue emitter, BD, was selected as a control reference. As illustrated in Figure 3c, the intense absorption band at 280–380 nm was attributed to π‐π^*^ and n‐π^*^ transitions of the DIDCz backbone, while a triple absorption band from 390 to 460 nm originated from the inherent *S*_0_‐*S*_1_ transition (corresponding to the calculated absorption of *S*_0_‐*S*_1_ transitions). The DIDCz‐tBu emitted a sharp emission band in toluene with a peak wavelength (*λ*_em_) at 460 nm and the Stokes shift was only 7 nm, which is smaller than most of narrowband ICz‐ or B/N‐based chromophores (Table S6, Supporting Information). Moreover, a slight spectral peak drift of 7.5 nm was observed in PL spectra upon of solvent polarity modulation. Generally, the Stokes shift and solvent effect demonstrate possible structural relaxations between excited and ground states. The rigid DIDCz skeleton with five‐membered interlocked structures minimizes most adverse molecular vibrations and molecular polarity changes. In the solid film state, 1% DIDCz‐tBu was doped in the anthracene‐based host 9‐(naphthalen‐1‐yl)‐10‐(naphthalen‐2‐yl)anthracene (BH) matrix with TTF assistants, where triplet excitons can be harvested via triplet up‐conversion. An outstanding PLQY of 96.5% was observed in the DIDCz‐tBu:BH film under the nitrogen atmosphere, which was nearly the same as the BD reference (94.4%). The large dipole strength contributes to achieving efficient quantum yields. As shown in Figure 3e, the transient photoluminescence (TrPL) exhibited single exponential decay with a prompt fluorescence lifetime (*τ*_p_) of 11.8 ns. Consequently, the singlet radiative rate constant (*k*_r_) for DIDCz‐tBu is 8.2 × 10^7^ s^−1^. The above consequences indicate that high efficiency and narrowband blue emission can be expected in the DIDCz‐tBu based EL devices.

### 2.3 EL Performances of the DIDCz‐Based Fluophosphor

**Figure 4:**
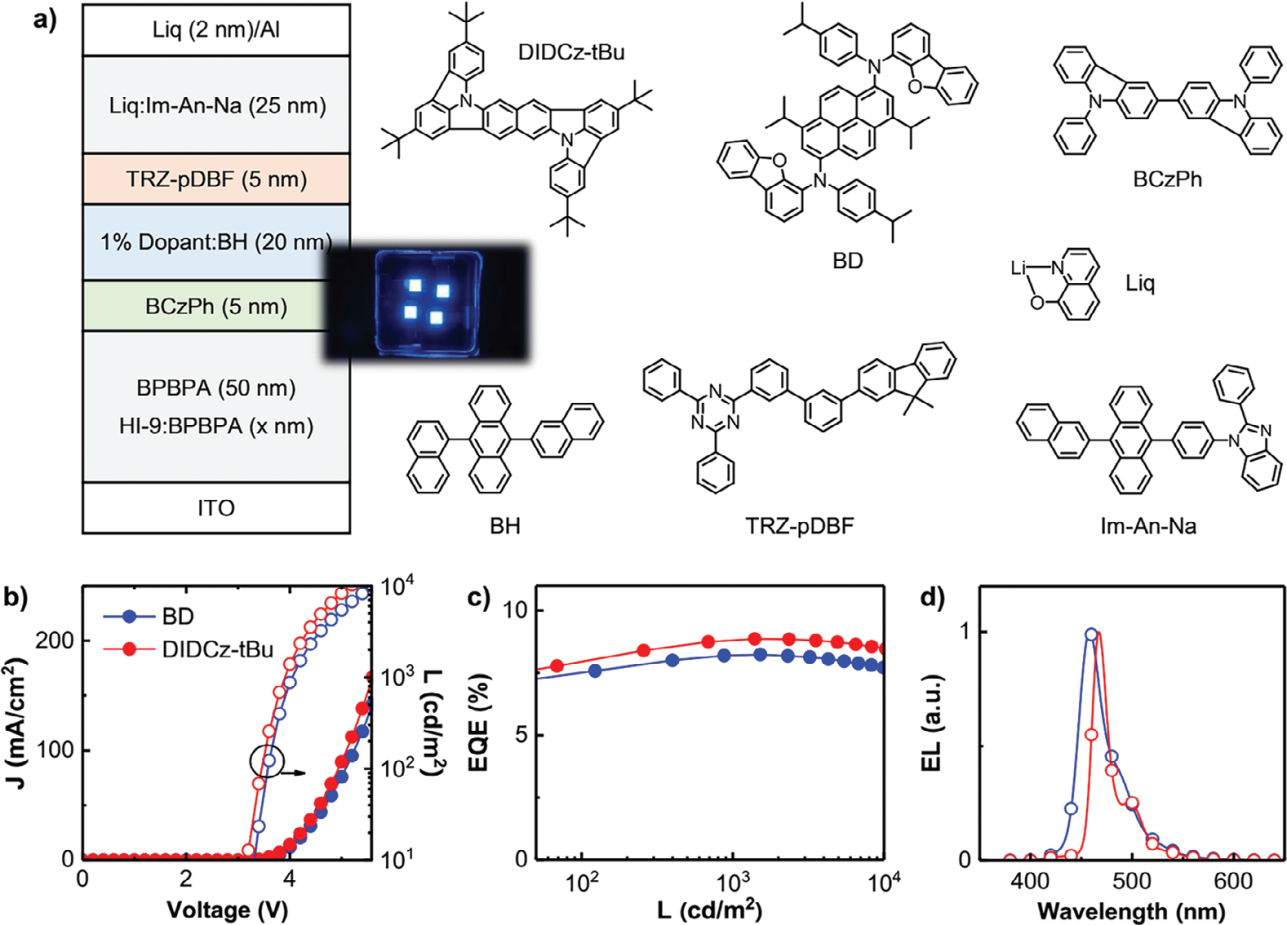
a) Device structure schematic of the DIDCz‐tBu based blue OLEDs and the chemical structure of organic functional materials. b) Current density‐voltage‐luminance (*J‐V‐L*) characteristics, c) External quantum efficiency versus luminance (EQE‐L) characteristics and d) EL spectra of the involved DIDCz‐tBu and BD based devices.

The superior photoelectric and narrowband spectral characteristics inspire us to further explore EL performances of DIDCz‐tBu emitters. Generally, EL spectral shape and the light coupling efficiency are close related to the optical microcavity effect from the transport layer cavity. The device structure was determined as ITO (95 nm)/3% HI‐9:N^4^,N^4^,N^4^″,N^4^″‐tetra([1,1′‐biphenyl]‐4‐yl)‐[1,1′‐biphenyl]‐4,4′‐diamine (BPBPA, *x* nm)/BPBPA (50 nm)/9,9′‐diphenyl‐9H,9′H‐3,3′‐bicarbazole (BCzPh, 5 nm)/1% DIDCz‐tBu:BH (20 nm)/2‐(3′‐(9,9‐dimethyl‐9H‐fluoren‐2‐yl)‐[1,1′‐biphenyl]‐3‐yl)‐4,6‐diphenyl‐1,3,5‐triazine (TRZ‐*p*DBF, 5 nm)/30 wt.% Lithium 8‐Hydroxyquinolinolate:1‐(4‐(10‐(naphthalen‐2‐yl)anthracen‐9‐yl)phenyl)‐2‐phenyl‐1*H*‐benzo[*d*]imidazole (Liq:Im‐An‐Na, 25 nm)/Liq (2 nm)/Al (100 nm). For comparative purposes, the BD dye was served as a control reference. The optimal hole injection layer (HIL) cavity was 75 nm, correlating with the highest EQE efficiency and blue index (Figure S11, Supporting Information). As shown in **Figure** **4**, DIDCz‐tBu‐based OLEDs achieved a maximum EQE efficiency of 8.87% with negligible efficiency trade‐off, compared to 8.24% for the BD‐based device. Similar to computed spectra, 0–1 and 0–2 vibrational EL peaks were weak, and thus, leading to a small FWHM of 18 nm and a CIE coordinate of (0.124, 0.146). The efficient and narrowband emission here validates the superiority of molecular vibration limited strategy. More importantly, DIDCz‐tBu‐based device revealed a long‐term T95 lifetime of 111 h under the current density of 50 mA cm^−2^, corresponding to 1545 h at the initial luminance of 1000 cd m^−2^. The operating lifetime was 14% longer than BD emitters, indicating higher chemical stability for the DIDCz‐based framework than those commercial pyrene‐based emitters. The EL achievements manifest one of the narrowest FWHM and longest operating life blue OLEDs among the reported PAH chromophores (Table S11, Supporting Information). In addition, DIDCz‐tBu emitter with top‐emitting structure was also fabricated on the reflected ITO/Ag/ITO anode. Under the optimized HTL thickness on a second cavity (Figure S13, Supplementary Information), the TB3 device showed a maximum current efficiency of 8.72 cd/A and high blue index of 115.5 with CIEy coordinate at 0.077.

As DIDCz‐tBu dyes have achieved good yields in both EQEs and operating lifetimes of TTF‐based OLEDs. Their TTF contribution and aging processes were investigated in further. EQE yields of DIDCz‐tBu based devices were much higher than conventional fluorescence (6.25%). Therefore, triplet utilizations from TTF process should participate in the exciton radiation. The emission from TTF (*I*_TFF_) is related to the Equation (1) below,

(1) $$\frac{1}{\sqrt{I_{\mathrm{TTF}}}}\propto A+\nu_{c}\cdot t$$

where *ν*_c_ is the decay rate of triplet excitons from TTF process and 1/A^2^ is defined as the TTF ratio. As shown in Figure S16 (Supporting Information), no TrEL overshoot was observed at the falling edge, confirming holes and electrons should not be trapped simultaneously or gathered in the emitting layer (EML). TrEL profiles possessed a thrice decay, with a nanosecond scale transient lifetime and two microsecond delayed fluorescence. With increase of current density, average delayed fluorescence lifetimes (*τ*_d_s) from TTF up‐conversion decreased gradually from 12.3 µs (at 1 mA cm^−2^) to 5.7 µs (at 100 mA cm^−2^). The TTF ratio improved to 34.2% at a high brightness region, indicating excitons from *T*_n_ states can efficiently convert into the *S*_1_ state. At the practical brightness of 1000 cd m^−2^, the TTF ratio has reached beyond 25%, giving a favorable efficiency of 8.62%. In addition, both DIDCz‐tBu and BD dyes observed high EQE_prompt_ of 6.67% and 6.79%, which is in accord with their PLQY yields. Thus, a small EQE efficiency improvement for DIDCz‐tBu emitters is mainly contributed to more efficient TTF up‐conversion (Figure S14c, Supporting Information). As displayed in Figure S20 (Supporting Information), the angle‐dependent PL spectrum of DIDCz‐tBu emitters exhibited a high horizontal dipole orientation (Θ_//_) value of 81.6 %, which is beneficial for improving the light out‐coupling efficiency.

Understanding the aging process is important to figure out the deteriorated source that attributes to the formation of exciton quenchers or carrier traps. As shown in Figure S15 (Supporting Information), The J‐V characters were nearly identical between pristine and aged DIDCz‐tBu devices with a very small voltage movement (less than 0.1 V), indicating that those carrier traps from excited states were negligible at the initial aging stage (from T100 to T90 state). Carrier traps can be further confirmed by capacitance‐voltage (C‐V) analysis. The parallel capacitance (*C*_P_) peak at the turn‐on voltage region represents the carrier injection and recombination process. As a result, the *C*_P_ peak onset was observed at voltage of 2.8 V, corresponding to the charge injection moment of the J‐V curve. The degraded device showed similar carrier injection peak at the voltage of 3.5 V with a maximum *C*_P_ of 3.3 nF (3.5 nF for a fresh device), illustrating that those carrier traps deterioration is minimal in the aging process. Comparison of TrEL profiles and J‐V characters at pristine and T90 stages were measured in Figure S16 (Supporting Information). The aged DIDCz‐tBu device showed degraded TTF ratio at low current densities with shorter delayed fluorescence lifetimes. The decay of prompt and delayed EQE yields were synchronized, suggesting generated non‐radiative quenchers was the main contributor for luminous degradation. In addition, compared to the BD emitter with weak C─N chemical bonds on the phenylamine groups, the DIDCz‐tBu dopant and the BH host contain only aromatic nucleus or multi‐N‐heterocycles on the molecular backbones. Their bond dissociation energies (BDEs) are high enough to resist degradation of molecules in the excited state or charged‐excited states (Figure S19 Supporting Information), which are higher than the BDEs of C─N bonds. Therefore, DIDCz‐tBu‐based TTF devices achieved satisfied operating stability in the optimized device configuration. The originate of non‐radiative quenchers should be resulted from the highly excited state in exciton‐exciton annihilation products (highly excited state energies can be over 5.6 eV), which is higher than BDEs of blue dyes and the host matrix.

Besides TTF mechanism, an alternative strategy to adopt triplet excitons is the TSF method, whereby the TADF material is adopted as a sensitizer for fluorophores. Given that an efficient Förster energy transfer between a sensitizer and the fluorophore is the prerequisite to promote TSF process, blue emitter of 5‐(3,11‐dimethyl‐5,9‐dioxa‐13b‐boranaphtho[3,2,1‐de]anthracen‐7‐yl)‐10,15‐diphenyl‐10,15‐dihydro‐5H‐diindolo[3,2‐a:3′,2′‐c]carbazole (*m*MDBA‐DI) was chosen as a TADF sensitizer.^[^ ^47^ ^]^ Thus, TSF devices were fabricated with the following configuration: ITO (95 nm)/3% HI‐9:BPBPA (60 nm)/BPBPA (30 nm)/BCzPh (10 nm)/3,3′‐di(9H‐carbazol‐9‐yl)‐1,1′‐biphenyl (mCBP, 15 nm)/EMLs/2,8‐bis(diphenylphosphineoxide)dibenzofuran (PPF, 10 nm)/Im‐An‐Na (30 nm)/Liq (2 nm)/Al (100 nm), where the EML contained 0.5% DIDCz‐tBu:20 wt.% *m*MDBA‐DI:PPF (*x* = 25, 30, 35 nm, devices TSF2‐TSF4). As shown in Figure S17 (Supporting Information), TSF2 device achieved an optimized EQE of 22.3% in maximum and 17.4% at a high brightness of 1000 cd m^−2^, showing a small efficiency roll‐off. The CIEy coordinate of 0.184 was nearly identical to TTF‐based devices with an emission peak at 468 nm. The T50 lifetime for DIDCz‐tBu‐based TSF device was 122 h. We believe that by further optimizing the device structures and developing more efficient blue sensitizers, EL performances of DIDCz‐tBu‐based TSF devices can be further improved (**Table** **2**).

**Table 2:** EL properties of the DIDCz‐tBu and BD‐based blue OLEDs.

Emitter	V_ON_ ^a)^ [V]	maximum	at 1000 cd m^−2^	EL Peak^b)^ [nm]	FWHM^b)^ [nm]	CIE [*x, y*]^b)^	T95 [h]
		CE/PE/EQE (cd/A, lm/W, %)	Voltage/CE/PE/EQE (V, cd/A, lm/W, %)				
BD	3.04	7.49/6.12/8.24	4.04/7.49/5.83/8.20	458	31	0.138, 0.103	97^c)^, 854^d)^
DIDCz‐tBu	2.86	9.79/8.42/8.87	3.88/9.75/7.90/8.80	467	18	0.125, 0.146	111^c)^, 1545^d)^
DIDCz‐tBu (TSF)	3.38	31.6/27.7/22.3	5.98/23.8/12.5/17.4	468	34	0.139, 0.184	122^e)^

^a)^ *V*_ON_ is defined as the driving voltage at a brightness of 1 cd m^−2^;

^b)^ Corresponding EL peak, FWHM, and CIE (*x, y*) were measured at luminance of 1000 cd m^−2^;

^c)^ The operating lifetime was measured at a current density of 50 mA cm^−2^;

^d)^ The operating lifetime at an initial luminance of 1000 cd m^−2^, using an acceleration factor of 1.7;

^e)^ The T50 lifetime at an initial luminance of 100 cd m^−2^.

## 3 Conclusion

In summary, we have developed narrowband and efficient blue multi‐N‐heterocycles by employing molecular vibration limited strategies in the extended π‐conjugated ICz skeleton. With advancements of SOS calculations, molecular vibration characteristics of the N‐*Na*‐N‐based fluorophores that contribute to spectral broadening can be classified. The interrelationships between ICz‐cyclization sites and spectral shape control were distinguished. As a result, compared to other cyclization patterns, 2,3,6,7‐ICz cyclization is the optimized choice for constructing narrowband N‐*Na*‐N‐based blue chromophores. Moreover, the vibrational emission peaks are highly depended on in‐plane swing of peripheral bonds (*Cz*‐1 vibration) and stretching behaviors of PAH frameworks (*Na*‐1, *Cz*‐2 and *Cz*‐3 vibrations) at the high molecular frequency region. By employment of 2,3,6,7‐ICz cyclization and steric hindrance substituents to manage inherent molecular resonances, the spectral shape of DIDCz‐tBu emitter become narrow with a small FWHM of 15 nm, showing reduced vibrational emission than other cyclization patterns or N‐*Ph*‐N‐based ICz fluorophores. The fabricated OLEDs achieved EQE efficiencies of 8.87% in the TTF fluorescence and 22.3% in the TSF device. Extremely stable T95 lifetime of 1545 h was exhibited in the TTF‐based device at an initial brightness of 1000 cd m^−2^, achieving one of the most narrowband and operational stable blue OLEDs among the reported PAH chromophores. Our investigation gives a new approach to manage molecular vibrations that contribute to spectral broadening, paving the way to design narrowband and pure blue multi‐N‐heterocycles.

## Conflict of Interest

The authors declare no conflict of interest.

## Supporting information



Supporting Information

## Data Availability

The data that support the findings of this study are available in the supplementary material of this article.
